# Supplementary material for: Near-term pathways for decarbonizing global concrete production
Source: Nat Commun. 2023 Jul 29;14:4574. doi: 10.1038/s41467-023-40302-0 (PMC10387082; doi:10.1038/s41467-023-40302-0)
Supplement: Supplementary file 1 — Supplementary Information [file 41467_2023_40302_MOESM1_ESM.pdf]

Near-term pathways for decarbonizing global concrete production

Josefine A. Olsson [a], Sabbie A. Miller [a,†], Mark G. Alexander [b]
[a] Department of Civil and Environmental Engineering, University of California, Davis
[b] Department of Civil Engineering, University of Cape Town
[†] Corresponding Author: T +1 530 754 6407, E sabmil@ucdavis.edu

This PDF file includes:

Supplementary Methods, pages S2 – S27
Supplementary Data Tables S1 – S8
Supplementary Figures S1 – S4
Supplementary References, pages S28 – S32

Other Supplementary Materials for this manuscript includes the following:

Supplementary Data 1 as archives:
Supplementary Data Sheet 1 Data for Figure 2
Supplementary Data Sheet 2 Data for Figure 3
Supplementary Data Sheet 3 Data for Figure 4
Supplementary Data Sheets 4-5 Data for Figures in the Supplementary materials PDF file

S.1. Methodology Summary

This work combines environmental impact assessment methods with proposed methods for quantifying the influence of various design decisions on the amount of concrete required for structural applications. The environmental impact assessments were used to assess greenhouse gas (GHG) emissions from concrete production, as well as the influence of several commonly discussed mitigation strategies proposed for reducing GHG emissions from concrete production. The GHGs considered in this work are CO_2, CH_4, and N_2O, and they were assessed in terms of CO_2-eq using the 100a global warming potentials from the Intergovernmental Panel on Climate Change [1]. The environmental impact assessments were performed on an initial cradle-to-gate scope; namely, GHG emissions from raw material acquisition, constituent processing, and through concrete batching were considered. Initial comparisons were drawn on a cubic meter basis. To account for the influence of design decisions, the effects on the quantity of concrete required for set applications, considering factors such as concrete strength, reinforcement ratio, and material longevity were incorporated. For each of these iterations, associated changes in concrete constituents and the influence of the environmental impacts of steel, which has notable GHG emissions from production [2], were addressed.

S.2. GHG Emissions and Mitigation at Cement / Concrete Production

S.2.1. Environmental impact modeling of cement and concrete

For this work, both process-based (e.g., emissions from calcination) and energy-based emissions (e.g., emissions from combusting fossil fuels for thermal energy) were considered to assess GHG emissions. The energy-based emissions were determined through both energy demands (e.g., incorporating equipment efficiency) and energy-resources (e.g., fuel types) and their combined effects on GHG emissions; emissions from fuels used in transportation were included as energy-based emissions. While the use phase was not directly a component of the environmental impact comparisons, it was considered through the inclusion of performance-based comparison methods in the subsequent sections. Specific modeling assumptions for individual concrete constituents, transportation, and batching were based on the OpenConcrete tool developed at the University of California Davis [3]. The OpenConcrete tool is an open-source modeling tool to determine environmental impacts of cement-based materials, and it allows for user-controlled inputs (e.g., energy resources, equipment efficiency, material transportation distances) to determine 11 impacts: GHG emissions, NO_X emissions, SO_X emissions, CO emissions, Pb emissions, VOC emissions, PM_{10} emissions, $PM_{2.5}$ emissions, water consumption, water withdrawal, and energy demand. Impacts are determined for a cradle-to-gate scope for a cubic-meter of concrete [4].

Energy demand for cement kilns was based on efficiencies by type from the Cement Sustainability Initiative Getting the Numbers Right Initiative (GNR) [5], using energy demand values reported for the world average in the year 2016. The baseline value was taken as the global average kiln energy demand for 2015. The electricity requirements for cement production were from the same report, again based on the kWh/kg reported of the world in the year 2015. For the kilns, the baseline thermal energy fuel mix used was based on the global average breakdown of fossil fuel demand, alternative fuel demand, and bio-derived fuel demand reported by GNR [5]. The relative fraction of different fuels within these categories were based on reporting from the Global Cement and Concrete Association [6].

All processes requiring electricity (including cement and the energy demands for all other materials and processes) were modeled as using the relative consumption of different energy resources to produce electricity globally. This electricity mix was based on data from the International Energy Agency [7].

To assess concrete mixtures with varying concrete constituents, GHG emissions from producing constituents or attributable to specific processes that would be an element of the environmental impacts of the concrete mixtures (e.g., natural pozzolans, batching) were tabulated (Table S.1). These constituents and processes were then be used to determine GHG emissions from production of concrete by being weighted based on the mass of constituents used in a given concrete mixture.
The baseline emissions used for the projection models were based on current data from fly ash and slag. Even though supplies of these two well-established industrial byproducts may decrease in the future we anticipate that other mineral additives (e.g., natural pozzolans) will contribute to similar performance; we note the availability of certain natural pozzolans is regional, but that there are a wide range of pozzolanic materials that could be used (e.g., tuff, calcined clays, agricultural byproducts) [8,9] The GHG emissions baseline based on current production is within 1% of the emissions if natural pozzolans were used in this model instead of fly ash.

Table S.1. Greenhouse gas (GHG) emissions by constituent or process

Constituent / Process	unit	Baseline impacts (kg CO_2-eq)
Ordinary Portland Cement (OPC)	per kg	8.72E-01
Natural Pozzolans (NP)	per kg	5.74E-03
Limestone (L)	per kg	5.74E-03
Plasticizing Admixtures	per kg	4.89E+00
Fly Ash (FA)	per kg	0.00E+00
Shale Ash (SA)	per kg	0.00E+00
Silica Fume (SF)	per kg	0.00E+00
Fine Aggregate	per kg	3.45E-03
Coarse Aggregate	per kg	5.27E-03
Blast furnace slag (GGBS)	per kg	3.73E-01
Calcined Clay (CC)	per kg	2.99E-01
Batching	per m3	1.41E+00
Transport, rail	per tkm	2.47E-05
Transport, truck	per tkm	1.69E-04
Batching water	per kg	0.00E+00

S.2.2. Implementation of GHG emissions mitigation strategies

To explore the influence of commonly discussed mitigation strategies that can be applied in cement and concrete manufacture, three implementations of such strategies are considered in this work: (1) improving equipment efficiency; (2) using lower-emitting energy resources in cement kilns; (3) using lower-emitting energy resources for the requisite electricity. For (1), the global average kiln efficiency was replaced by that for more efficient preheater/precalciner kilns based on energy demand reported by GNR [5]. For (2), oil energy resources were modeled as replacing higher emitting energy resources in the cement kilns. For (3), wind electricity was modeled as replacing the higher emitting energy resources for all electricity demand.

S.2.3. Concrete mixtures and comparisons on a volume basis

Using a database compiled from the literature, using concrete mixtures from several data sources: [10–30]. These papers were used to assemble a set of 372 concrete mixtures with various material properties, see Supplementary Data Sheet 1, (note: strength for these mixtures was adjusted using the methods stipulated by [31] to account for differences in test specimen dimensions). These papers were selected because they all use CEM I type or ASTM Type 1 cement, which has a relatively consistent level of minerals blended with clinker. These types of cements in the papers were also blended with certain

levels of mineral additives (e.g., FA, GGBS), which allowed for more accurate modeling of GHG emissions due to known ratios of mineral additive to clinker content.

To plot box and whisker plots of the GHG emissions, binder content, and clinker content variability by strength group, mixtures were organized by strength. Mixtures within ± 3 MPa of the intended strength (20, 35, and 50 MPa) were used to examine the before mentioned distributions. The role each of the manufacturing-stage GHG mitigation strategies have on trends for GHG emissions by strength group were plotted as well.

S.3. GHG Mitigation for Member and Structure Design (excluding longevity considerations)

S.3.1. Relationships between environmental impacts and concrete strength

To derive functions that relate GHG emissions per cubic meter of concrete produced to the compressive strength specified, equations derived by Fan and Miller were applied [32]. Due to their varying effects on strength development and GHG emissions, different parameters were used in these equations for each mineral additive considered in this work: NP, L, GGBS, FA, SF, SA, and CC. These values were based on fitting parameters to experimental data presented by [11,14,15,21,23]. The mixtures from these literature sources were selected because the mixtures tested facilitated comparisons across several water-to-binder ratios as well as several replacement ratios of ordinary Portland cement (OPC), and due to the consistency in mixture constituents achieved by using experimental protocols from a limited set of authors.

The parameters used to relate concrete constituents to GHG emissions are shown in Table S.2. These fitting parameters are based on the method and nomenclature presented by Fan and Miller [32], which is as follows:

$$i_1 = k_A \bullet c + k_B \quad (1)$$

$$i_2 = k_C \bullet s + k_D \quad (2)$$

$$i_3 = i_1 + i_2 \quad (3)$$

where i_1 is the environment impact of a cubic meter of concrete containing no mineral additives for a known cement content, c, i_2 is the environmental impact from the use of a known quantity of mineral additive, s, for a cubic meter of concrete, k_A is a constant that depends on cement manufacturing, k_B is a constant that depends on remaining materials and production for the concrete mixture when no mineral additives are present, k_C is a constant that depends on the mineral additive, k_D is a fitting parameter to account for differences in materials and production for the concrete mixture when mineral additives are present. Here, we fit k_A and k_B parameters from mixtures containing OPC as the only cementitious binder from literature sources with varying binary blends (e.g., with OPC and natural pozzolans). This variation in literature sources has accompanying variation in binder contents, which leads to the minor differences in these parameters as reported in Table S.2.

Table S.2. Parameters to relate concrete constituents to greenhouse gas (GHG) emissions per cubic meter of concrete.

Mineral Additive	k_A	k_B	k_C	k_D
Limestone	0.88	35.41	0.036	-0.0099
Natural Pozzolans	0.88	35.41	0.032	-0.1224
Shale Ash	0.88	35.41	0.169	-0.0499
Calcined Clay	0.88	35.41	0.353	-0.0713
Silica Fume	0.88	35.41	0.295	0.0721
Fly Ash	0.87	29.89	0.147	-0.0856
Blast Furnace Slag	0.86	37.42	0.265	0.2101

To extend these relationships to capture concrete compressive strength, again, a method presented Fan and Miller was applied [32]. This method builds from Abram's law, in which compressive strength (f_c) is correlated to the water-to-binder ratio (*w/b*) for concrete mixtures:

$$f_c = \frac{k_1}{k_2^{w/b}} \tag{4}$$

where k_1 and k_2 are fitting parameters, and the other parameters are as previously defined; here w/b is considered on a weight basis. These fitting parameters were determined for the mixtures reported in [11,14,15,21,23] for each reported ratio of mineral additive to OPC. These parameters are presented in Table S.3. A limited number of sources has been selected intentionally to maintain consistency in mixture design, including type of cement, how mineral additives were integrated into the paste, and consistent degrees of water use. We note that while many additional permutations can be made of concrete mixtures; this consistency facilitates robust modeling.

Table S.3. Parameters to relate water-to-binder ratio. OPC = Ordinary Portland Cement.

Mineral Additive	*Mineral additive to OPC ratio*	k_1	k_2
Limestone (L)	8.18E-01	1.72E+02	6.85E+01
	5.38E-01	1.26E+02	2.13E+01
	3.33E-01	1.35E+02	1.53E+01
	1.76E-01	1.37E+02	1.11E+01
	0.00E+00	1.41E+02	9.72E+00
Natural Pozzolans (NP)	8.18E-01	1.26E+02	2.29E+01
	4.29E-01	1.45E+02	1.84E+01
	1.76E-01	1.34E+02	1.24E+01
	0.00E+00	1.41E+02	9.72E+00
Shale Ash (SA)	6.67E-01	1.25E+02	1.66E+01
	4.29E-01	1.31E+02	1.38E+01
	2.50E-01	1.42E+02	1.23E+01
	1.11E-01	1.41E+02	1.01E+01
	0.00E+00	1.41E+02	9.72E+00
Calcined Clay (CC)	3.33E-01	2.19E+02	1.50E+01
	2.50E-01	2.15E+02	1.46E+01
	1.76E-01	2.08E+02	1.44E+01
	1.11E-01	1.84E+02	1.25E+01
	5.26E-02	1.71E+02	1.19E+01
	0.00E+00	1.41E+02	9.72E+00
Silica Fume (SF)	2.50E-01	2.16E+02	1.28E+01
	1.76E-01	2.06E+02	1.23E+01
	1.11E-01	1.99E+02	1.23E+01
	5.26E-02	1.76E+02	1.14E+01
	0.00E+00	1.41E+02	9.72E+00
Fly Ash (FA)	5.81E-01	1.63E+02	1.90E+01
	5.00E-01	1.63E+02	1.57E+01
	4.23E-01	1.62E+02	1.33E+01
	3.31E-01	1.49E+02	1.04E+01
	2.50E-01	1.46E+02	9.21E+00
	1.49E-01	1.35E+02	7.76E+00
	0.00E+00	1.38E+02	8.42E+00
Blast Furnace Slag (GGBS)	1.57E+00	1.85E+02	4.31E+01
	1.29E+00	1.92E+02	3.01E+01
	1.00E+00	1.79E+02	1.91E+01
	7.14E-01	1.59E+02	1.20E+01
	4.29E-01	1.29E+02	7.67E+00
	2.14E-01	1.12E+02	6.51E+00
	0.00E+00	1.15E+02	6.70E+00

S.3.2. Environmental impacts of reinforced concrete members

Building from the terms derived for GHG emissions as a function of concrete constituents, relationships were developed to examine the role of concrete strength and reinforcement ratio on the environmental impacts of designed members. Equations to calculate the environmental impact of reinforced concrete members were based on the method developed by Kourehpaz and Miller [33] for design of (square) columns subjected to an axial load and slabs in bending for the design stages cracking, yielding and ultimate according to ACI-318 [34]. The equations were converted into Eurocode 2 [35] and the Indian Standard [36] to facilitate comparisons. Using concrete cylinder compressive strength, as well as steel properties of 420 MPa yield strength, GHG emissions of 1.03 kg CO_2-eq/kg (lower range) and 2.29 kg CO_2-eq/kg (upper range) [37], approximate GHG emissions from transportation of 0.108 kg CO_2-eq/kg

[33], and density of 7800 kg/m^3, the following parameters were defined to construct necessary relationships for designed members:

- l = column height/slab span [m]
- b = column width/slab unit-width [m]
- f'_c = design compressive strength of concrete [MPa]
- h = column width/slab thickness [m]
- w = applied uniform load [kN/m]
- f_r = rupture strength of concrete [MPa]
- A_s = reinforcement area [m^2]
- f_y = yield strength of steel reinforcement [MPa]
- F_{conc} = applied axial load (column) [kN]
- i_s = volumetric environmental impact - steel [CO_2-eq/m^3]
- i_c = volumetric environmental impact - concrete [CO_2-eq/m^3]

A standard height of 3.5m was used for the column and the span length for the slab was 7m. The authors note there is a minor range in densities possible for steel reinforcement, and our models suggest less than 1% difference in GHG emissions findings would occur for the column and slab modeled if density of reinforcing steel were to increase by 50 kg/m^3.

The environmental impact equation for a general reinforced concrete member according to Kourehpaz and Miller [33] was given as:

$$I_{RC} = l * (bh - A_s) * i_c + A_s l i_s \quad (5)$$

where I_{RC} refers to the environmental impact of a reinforced concrete member, determined based on required cross-sectional area to sustain the load and the volumetric impact of steel and concrete, and other terms are as defined above. This equation was adapted to reflect concrete building codes by setting: (a) the maximum force to not exceed the force that can be withstood by the concrete and steel in a column; or (b) setting the maximum moment to not exceed the moment for slabs designed for cracking, yield, or ultimate stages of the moment-curvature relationship. In each case, the member dimension "h" in Equation 5 was allowed to vary for different concrete strengths and reinforcement ratios under a constant load, but other dimensions were held constant. For the slabs considered in this work, the members were assumed to be simply supported flat slabs and uniformly loaded. The models here examine the slab as a 1m wide beam. The equations used to relate environmental impacts to these varying parameters for column design, slab design for cracking-stage, yield-stage, and ultimate-stage for each of the three considered design codes were derived as follows:

S.3.2.1. Environmental impact relationships based on the United States ACI-318 code

Note: For the purpose of this analysis, the concrete compressive strengths, f'_c, and steel tensile strength, f_y, are assumed to be factored design strengths.

To relate environmental impacts to concrete compressive strength and the area of steel for a reinforced concrete column design, the following relationship was derived:

$$I_{column} = l * (F_{conc} - f_y A_s) * \frac{i_c}{f'_c} + A_s l i_s \quad (6)$$

For this scenario, the allowable reinforcement ratio was defined as:

$$0.01 \leq \frac{A_s}{A_{section}} \leq 0.08 \quad (7)$$

For a slab designed at the cracking stage, the following relationship was derived:

$$I_{crack} = 1.1 l^2 \sqrt{wb} \left(\frac{i_c}{f'^{0.25}_c} \right) - A_s l i_c + A_s l i_s \quad (8)$$

where the thickness of the member is related to the load, the width (1m unit-width), and the rupture strength of the concrete through the equation:

$$h = \sqrt{\frac{6}{8} \frac{w l^2}{f_r b}} \quad (9)$$

and the rupture strength is related to the compressive strength using the equation:

$$f_r = 0.62 \sqrt{f'_c} \quad (10)$$

which allows for the determination of the critical moment, M_{cr}:

$$M_{cr} = \frac{f_r b h^2}{6} \quad (11)$$

For these slabs, the allowable reinforcement ratio was set using:

$$0.002 \leq \frac{A_s}{A_{section}} \quad (12)$$

The equation for a beam in bending was adapted for a reinforced concrete slab at the rebar yielding stage as follows:

$$I_{yield} = 0.67 l A_s f_y \frac{i_c}{0.7 f'_c} + l \left(\frac{w l^2 b}{8 A_s f_y} + bm - A_s \right) i_c + A_s l i_s \quad (13)$$

where moment was derived using the following equations according to the ACI code:

$$M_y = A_s f_y \left(d - 0.67 \frac{A_s f_y}{0.7 f'_c b} \right) \tag{14}$$

and, considering a simply supported slab, the allowable moment is determined as for a simply supported beam:

$$M_{max} = \frac{wl^2}{8} \tag{15}$$

Note that 70% of the compressive strength, f'_c, is assumed when the rebar starts yielding.

For a reinforced concrete slab designed at the ultimate stage of the moment-curvature relationship, the environmental impact of the designed member as it relates to the material properties, material volumes, and material environmental impacts was defined as follows:

$$I_{ultimate} = 0.59 l A_s f_y \frac{i_c}{f'_c} + l \left(\frac{wl^2 b}{8 A_s f_y} + bm - A_s \right) i_c + A_s l i_s \tag{16}$$

where the moment was defined as:

$$M_u = A_s f_y \left(d - 0.59 \frac{A_s f_y}{f'_c b} \right) \tag{17}$$

and the same maximum moment in Equation 15 was applied. The ACI 318 code uses a strength reduction factor of 0.9 for members in bending, at the ultimate stage (LRFD), per Section 21.2.1.

S.3.2.2. Environmental impact relationships based on the Eurocode2 code

Note: For the purpose of this analysis, the concrete compressive strengths, f'_c, and steel tensile strength, f_y, are assumed to be factored design strengths.

For the column design, the general relationship between member environmental impact and the design parameters considered remained the same as that for the ACI code:

$$I_{column} = l * (F_{conc} - f_y A_s) * \frac{i_c}{f'_c} + A_s l i_s \tag{18}$$

However, the definition for the allowable reinforcement ratio differed, and it was defined as follows:

$$max \left(0.1 \frac{F}{f_y A_{section}}, 0.002 \right) \leq \frac{A_s}{A_{section}} \leq 0.04 \tag{19}$$

For the design of a reinforced concrete slab at the cracking stage, the relationship between the volume of concrete required, volume of steel, and material strengths as they informed the member's environmental impact differed slightly from the ACI code. The new relationship was derived as follows:

$$I_{crack} = 1.6l^2\sqrt{wb}\left(\frac{i_c}{f'^{0.33}_c}\right) - A_s l i_c + A_s l i_s \tag{20}$$

where the member thickness was defined as:

$$h = \sqrt{\frac{6}{8}\frac{wl^2}{f_{ctm}b}} \tag{21}$$

and the relationship between concrete tensile strength, f_{ctm}, and compressive strength is given as:

$$f_{ctm} = 0.30f'^{0.67}_c \tag{22}$$

which allows for the determination of the critical moment, M_{cr}:

$$M_{cr} = \frac{f_{ctm}bh^2}{6} \tag{23}$$

For this member, the allowable reinforcement ratio was defined, based on Eurocode2, Section 9.2.1.1 [35], as:

$$max\left(0.26\frac{f_{ctm}}{f_y}, 0.0013\right) \leq \frac{A_s}{A_{section}} \leq 0.04 \tag{24}$$

For a slab in bending designed at the yielding stage, the same equation as applied for ACI 318 also applied for the Eurocode2, namely:

$$I_{yield} = 0.67lA_s f_y \frac{i_c}{0.7f'_c} + l\left(\frac{wl^2b}{8A_s f_y} + bm - A_s\right)i_c + A_s l i_s \tag{25}$$

Further, using the following equations to relate yield moment to the other parameters measured in this work:

$$M_y = A_s f_y\left(d - 0.67\frac{A_s f_y}{0.7f'_c b}\right) \tag{26}$$

Note that 70% of the compressive strength, f'_c, is assumed when the rebar starts yielding. And again where, for a uniformly loaded simply supported slab, the maximum moment would be defined as:

$$M_{max} = \frac{wl^2}{8} \tag{27}$$

For a slab in bending designed for the ultimate stage, the Eurocode design code, again, led to moderate differences from the ACI code in how the environmental impacts of the member would relate to its constituents and their properties. Namely, the environmental impact equation would be defined as:

$$I_{ultimate} = 0.51lA_sf_y\frac{i_c}{f'_c} + l\left(\frac{wl^2b}{8A_sf_y} + bm - A_s\right)i_c + A_sli_s \tag{28}$$

For this equation, the moment would be defined as:

$$M_u = A_sf_y\left(d - 0.51\frac{A_sf_y}{f'_cb}\right) \tag{29}$$

where again, the maximum moment would be defined based on Equation 27. The Eurocode uses partial safety factors of 1.5 for concrete and 1.15 for reinforcing steel at the ultimate stage, per Section 2.4.2.4.

S.3.2.3. Environmental impact relationships based on the Indian Standard 456:2000 Code

Note: The Indian Standard uses cubic strength, 1.25 factor converts to cylinder strength, according to the Indian Standard (IS). Compressive strengths used are converted from cubic to cylinder strengths. For the purpose of this analysis, the concrete compressive strengths, f'_c, and steel tensile strength, f_y, are assumed to be factored design strengths.

As with the previously discussed codes, for the Indian Standard code, the equation that would allow for comparison of environmental impacts based on concrete and steel mechanical strengths and volumes can be defined as:

$$I_{column} = l * (F_{conc} - f_yA_s) * \frac{i_c}{f'_c} + A_sli_s \tag{30}$$

For the IS code, an appropriate reinforcement ratio for a column design would be defined, based on IS 456:2000, Section 26.5.3.1 [36], as:

$$0.008 \leq \frac{A_s}{A_{section}} \leq 0.06 \tag{31}$$

In the case of a slab designed at the cracking stage, the environmental impact relationship is similar to that found for the EuroCode2:

$$I_{crack} = 1.0351l^2\sqrt{wb}\left(\frac{i_c}{(f'_c)^{0.25}}\right) - A_sli_c + A_sli_s \tag{32}$$

For this case, again the thickness of the slab would be defined as:

$$h = \sqrt{\frac{6}{8}\frac{wl^2}{f_r b}} \tag{33}$$

where the relationship between concrete tensile rupture strength, f_r, and compressive strength is given, based on IS 456:2000, Section 6.2.2, as:

$$f_r = 0.7\sqrt{f'_c} \tag{34}$$

Using the stipulated values, the appropriate reinforcement ratio would be defined, based on IS 456:2000, Section 26.5.2.1, as:

$$0.0015 \leq \frac{A_s}{A_{section}} \leq 0.02 \tag{35}$$

For a slab in bending designed at the rebar yielding stage, a different equation than the prior two codes would be implemented. Namely, the following equation was derived:

$$I_{yield} = 0.67lA_s f_y \frac{i_c}{0.7f'_c} + l\left(\frac{wl^2 b}{6.96 * f_y} + bm - A_s\right)i_c + A_s l i_s \tag{36}$$

Here, as was done for the prior two codes presented, the moment relationship can be written as:

$$M_y = A_s f_y\left(d - 0.67\frac{A_s f_y}{0.7f'_c b}\right) \tag{37}$$

Note that 70% of the compressive strength, f'_c, is assumed when the rebar starts yielding. And again, where for a uniformly loaded, simply supported flat slab would have a maximum moment defined as:

$$M_{max} = \frac{wl^2}{8} \tag{38}$$

For a slab in bending designed at the ultimate stage, the relationship between the parameters investigated in this work and the total environmental impact of the slab would be defined as:

$$I_{ultimate} = lA_s f_y \frac{i_c}{0.67f'_c} + l\left(\frac{wl^2 b}{8A_s f_y} + bm - A_s\right)i_c + A_s l i_s \tag{39}$$

where the moment would be based on the relationship:

$$M_u = A_s f_y\left(d - \frac{A_s f_y}{0.67f'_c b}\right) \tag{40}$$

and the maximum moment given in Equation 38. At the ultimate stage, the compressive strength of concrete shall be assumed to be 0.67 times the characteristic strength. In addition, the Indian Standard uses partial safety factors of 1.5 for concrete and 1.15 for reinforcing steel, per Section 38.1.

S.3.3. Extension of member design to designed structures

In order to derive a proxy for how the specification of concrete mixture proportions and the decisions made in concrete component design could influence the built environment, a simplified set of extensions were derived. Namely, a system of equations for assessing the use of concrete in civil infrastructure and in buildings was determined. While the uses of concrete can be quite varied in civil infrastructure, an approach was not devised to estimate how design could influence roads and highways, which can be a large consumer of infrastructure concrete [38]. Rather, design considerations were focused on buildings.

For concrete buildings, a slightly more intricate set of equations was devised. Namely, for these structures, a system of columns and slabs were used to estimate the effects of design. In this case, the effects of single- or multiple-story structures were assessed to incorporate not only the effects of concrete strength and reinforcement ratio, but also the effects of increased deadloads with a greater number of stories. These influences were incorporated into this analysis through a method posited by Schmidt et al. [39]. In this work, the contributions of varying foundation and roofing materials were excluded from analysis, but they could lead to notable effects for large structures. The effects on building height and weight on lateral design in seismic regions were also not considered or discussed in this work.

To address concrete strength, reinforcement ratio, and the influence of whether buildings were built with multiple stories or a single story, several inputs were used. These included:

- A repeating building unit was used that consisted of 1 slab and 4 columns supporting it.
- The range of compressive strength for concrete considered was between 20 – 40 MPa
- The steel properties were the same as those for the reinforced concrete members discussed in Section S.3.2.
- The environmental impact for concrete as a function of its strength were based on the equations discussed in Section S.3.1. When no mineral additives were used, the parameters for the limestone mixtures at a 0% mineral additive replacement of OPC ratio were used. (Note: while the parameters for all mixtures with a 0% mineral additive replacement ratio had similar parameters, because some inputs used fits, the parameters are not identical. The use of the parameters based on the mixtures with 0% limestone were used as limestone is one of the most prevalent mineral additives used globally [5]).
- 20.3% mineral additive content in the cementitious materials based on the global average in 2015 [5]
- Water content, w= 185 kg/m^3
- Force = (2370 kg/m^3)(20m × 7m × 0.14m)/4 = 116 kN, assumed applied axial load from concrete slab self-weight for each column; the weight of columns themselves was neglected.

This work determined the role of design inputs for the concrete columns and the required column area, considering single vs multiple story units for varying compressive strength. To do this, again, a standard column height, l, of 3.5 m was consistently applied. The required column cross-section area was defined

based on:

$$A_{concrete} = \frac{F}{f'_c(1 + (N - 1) * \rho_{min})} \tag{41}$$

Where minimum reinforcement ratio, $\rho_{min} = 0.01$, is assumed when designing according to ACI-318. N is the ratio between the Young's modulus for steel and concrete, equal to E_s/E_c, and E_s is the modulus of steel, which is set here as 207 GPa; E_c is the modulus of concrete, which is calculated here as $4700\sqrt{f'_c}$, $22\left(\frac{f'_c}{10}\right)^{0.3}$, and $5000\sqrt{f'_c}$ for ACI-318, EC2 and the Indian Standard, respectively.
Also, $A_{concrete}$ is the cross-sectional area of the concrete column, F is the applied force, and f'_c is the compressive strength of the concrete. The total concrete area required for n single story (i.e., unstacked) units, $A_{total,unstacked}$, was given as:

$$A_{total,unstacked} = n * A_{concrete} \tag{42}$$

Then, the total concrete area required for n stacked units (i.e., multi-story with S being the number of stories) is given by:

$$S = n * \frac{(n + 1)}{2} \tag{43}$$

$$A_{total,stacked} = S * A_{concrete} \tag{44}$$

where $A_{total,unstacked}$, is the total concrete area for the columns in the stacked building. Cumulatively, the environmental impact for either the single- or multi-story units can be written as:

$$I_{col,unstacked} = l * (A_{total,unstacked} - 0.01 * A_{total,unstacked}) * i_c + l * 0.01 * A_{total,unstacked} * i_s \tag{45}$$

and

$$I_{col,stacked} = l * (A_{total,stacked} - 0.01 * A_{total,stacked}) * i_c + l * 0.01 * A_{total,stacked} * i_s \tag{46}$$

respectively.

To capture the influence of the slab in the repeating unit, the slab volume for varying compressive strength was defined using a similar structure as discussed previously. Here, again, a simply supported, one-way flat slab was assumed. It was modeled as having a constant length, l, of 7 m and a constant width, b, of 20 m. The concrete cover was modeled as 0.040 m. To reflect design requirements, constraints were checked in accordance with ACI 318 design code [40]. The required reinforcement ratio, ρ, for varying compressive strength was defined as:

$$\rho = 0.002 \tag{47}$$

The requisite slab thickness, h, to meet allowable deflection was then specified as:

$$h = \frac{l}{16} \tag{48}$$

Note: this deflection criterion is for ACI 318, for Eurocode2 the criterion for slab thickness limit for deflection control is $l/20 < d < l/14$ (where d is the effective depth, not the slab thickness), and for IS slab thickness limit for deflection control is $l/20 < d$.

Using these relationships, the area of reinforcing steel A_s, was then written as:

$$A_s = \rho * h * b \tag{49}$$

The external moment on the slab, M_n, was defined based on the loading and boundary conditions as:

$$M_n = \frac{8000 * l^2}{8} \tag{50}$$

Using these parameters, the effective slab depth, d, was then written as:

$$d = \frac{M_n}{A_s f_y} + \frac{0.59 A_s f_y}{f'_c b} \tag{51}$$

Values for the slab depth were then checked against the requirement and reinforcement ratio limits according to ACI-318. Namely, it was confirmed that the design met the following:

$$\frac{l}{16} < (d + m) < \frac{l}{10} \tag{52}$$

and

$$0.002 < \frac{A_s}{bd} \tag{53}$$

The environmental impact for the slab for varying compressive strength was calculated using equation 16.

With these inputs, the total environmental impact for the multi-storey units, $I_{total,stacked}$, and for the single-story units, $I_{total,unstacked}$, for varying compressive strength were then defined as:

$$I_{total,stacked} = 4 * I_{col,stacked} + I_{slab} \tag{54}$$

and

$$I_{total,unstacked} = 4 * I_{col,unstacked} + I_{slab} \tag{55}$$

respectively.

For Eurocode 2 and Indian Standard, the same equations were used to calculate the required column area but with different minimum reinforcement ratio. Namely, for Eurocode 2, the minimum reinforcement ratio was taken as per equation 19; for the Indian Standard, the minimum reinforcement ratio was taken as 0.008.

For the slab, again some differences were present for the Eurocode 2 and the Indian Standard relative to the above equations based on the ACI code. Namely, for the slab, the following minimum reinforcement ratios were used based on the Eurocode 2:

$$max\left(0.26\frac{f_{ctm}}{f_y}, 0.0013\right) \quad (56)$$

and the Indian Standard:

$$0.0015 < \frac{A_s}{bd} \quad (57)$$

Additionally, the required slab depth was calculated using equation 28 (Eurocode 2) and equation 39 (Indian Standard).

S.4. GHG Mitigation from Durability and Use of SCMs

The design life of concrete structures is largely governed by their durability. Current design codes are largely deficient in their ability to quantify design life in relation to the dominant deterioration mechanisms, since they rely on prescriptive rather than performance principles [41]. Codes also often conflate durability with concrete compressive strength, using strength as a proxy for durability. Research has shown that this premise is false [42]; durability is more related to the transport properties (penetrability) of concrete than to its strength, and with a range of SCMs now available, their different chemical performance can be used to advantage even when they display similar compressive strength.

Deterioration due to steel corrosion is the dominant mode of reinforced concrete deterioration. Provided that the various phases can be understood and scientifically described, design life can be quantified and controlled mechanistically, including within a probabilistic framework. Extending the service life of a concrete structure through proper durability design is possible with current knowledge. Other than corrosion of the reinforcing steel, other forms of deterioration exist which can be damaging, such as alkali-aggregate reaction. However, for corrosion, environmental chlorides (e.g., from marine salts or from the de-icing salts) are usually the most aggressive and pernicious problem. Carbonation of the concrete cover can also induce steel corrosion, but this is less pronounced than chlorides; here, a common misconception is that carbonated structures inevitably corrode, but other corrosion-inducing factors of moisture and oxygen must also be present, and frequently, provided the cover to steel is adequate, corrosion does not ensue [43]. Also, the majority of concrete in a building is indoors and protected from the weather and corrosive agents, and many facades are also protected.

S.5. GHG Mitigation from Different Service-Life Spans

S.5.1. Historical cement and cement-based material production and longevity

In order to address both GHG emissions from the historical global production of cement and cement-based composites, as well as the effects of increasing concrete in-use longevity, several existing models were implemented concurrently. First, global cement production data, by country or locality, were collected from the United States Geological Survey (USGS), capturing production from 1931-2015 (data from [47–70]). To examine in-use stocks, which were used to understand the effects of elongating concrete use-phase life period, the model derived by Cao et al. [71] was implemented. This implementation involved using that model's split ratios between residential, non-residential buildings and infrastructure, as well as in-service lifetimes for concrete structures. Import and export data necessary to convert production data from the USGS to consumption data needed to use by Cao et al.'s model were from the United Nations Comtrade database [72].

The GHG emissions to produce cement and concrete historically were adapted from the calculations presented in Section S.2, which reflect production in 2015. To capture historic cement production, these models were extended to reflect 1990, 1995, 2000, 2005, and 2010 production statistics. Namely, the global demand for different SCMs as well as the electricity required to produce cement and kiln efficiency in each of those years were based on [5]. The fraction of fossil fuels, waste fuels, and biomass used for thermal energy in the cement kilns were from [5]. However, due to limitations in available data, to increase data granularity beyond those general fuel categories, the relative fraction of different fuels within those groups (e.g., coal versus oil in the fossil fuels used) were all based on the relative fractions used in the same reference year, 2018, reported by Global Cement and Concrete Association [6]. The electricity mix for each of these years was based on global data from the International Energy Agency [7].

To extend these calculations to capture concrete production, several additional calculations were performed. The energy demand for other constituents and processes were based on values reported in Section S.2 and the energy grids were based on data from the representative year, using the same grids as were applied for cement production. To capture concrete constituents required, distributions of varying constituent inputs were fit to the data presented in Supplementary Data Sheet 1 for each of four strength classes: (i) < C16/20; (ii) C16/20 – C25/30; (iii) C25/30 – C35/37; (iv) > C35/45. Fractions of use by strength category for each year were based on statistics reported by the European Ready Mixed Concrete Organization (ERMCO) [73–76]. Because these fractions were only reported for some countries/regions, a global fraction of concrete consumption by strength class was estimated by using a weighted fraction by national production statistics reported by the USGS (using data from the representative years, collected as discussed above). The ERMCO reports were also used to collect data on the relative fraction of cement used in concrete, with the exception of data for China, which were based on [77]. While cement produced, but not used in concrete, could be used in several applications, this remaining cement was modeled herein as mortar (using an assumption applied in prior publications [71,77,78]). The distributions of constituents required for mortar production were based on the relative fractions of cement, masonry cement, lime, sand, and water used in the American Standards for Testing Materials (ASTM) protocol [79]. Fractions of mineral additives were based on two factors. The first incorporated the relative quantity of limestone and other fillers reported for masonry cement by the Portland Cement Association (PCA) [80]. All masonry cement modeled based on the ASTM code was

assumed to have the same average mineral filler reported by the PCA. In addition to this quantity mineral additive, the second input to mineral additives was that all cement was assumed to have the same general ratio of SCMs as reported above, based on global averages for the years reported.

S.5.2. Historical cement-based material production emissions

To capture full historical emissions profiles for cement and concrete, values were extrapolated from the reference years assessed. Namely, linear interpolation of emissions profiles was applied to years that fell between 1990, 1995, 2000, 2005, 2010 and 2015. For all years prior to 1990, emissions were modeled as having the same emissions per kg of cement consumed as was modeled in 1990.

To apply these emissions profiles to other models used in this work, the kg GHG emissions / m^3 concrete ($\rho_{GHG\ emissions}$) was used. This emissions per cubic meter of cement-based material reflects a global average. To apply this factor to historical global cement production, first the mass of cement produced globally was summed annually from the individual nations reported by the USGS (discussed above). Using the distributions for constituents used in cement and concrete discussed in Section S.5.1., for every kg of cement used, there are on average 7.3 kg of cement-based materials. This is to say, for every 1 kg of cement used, there are approximately 6.3 kg of other constituents (e.g., water, aggregates) used. For example, for a 2,400 kg/m^3 concrete mixture, approximately 330 kg of cement and 2070 kg of other constituents are used. The majority of cement produced goes to the production of concrete, but production of other materials, like mortar, is incorporated too. The average density of cement-based materials ($\rho_{concrete}$) is 2370 kg/m^3 from these distributions. Using these inputs, the mass of cement produced (m_{cement}) was converted to a volume of cement-based materials ($V_{concrete}$) based on:

$$V_{concrete} = m_{cement} \frac{m_{concrete}}{\rho_{concrete}} \tag{58}$$

With this value for the volume of concrete produced, the world GHG emissions per year ($m_{GHG\ emissions}$) was determined as a function of the volume of cement-based materials produced globally and the emissions assessed in kg GHG emissions / m^3 concrete:

$$m_{GHG\ emissions} = V_{concrete} * \rho_{GHG\ emissions} \tag{59}$$

S.5.3. GHG emissions reductions as from changes in longevity

The influence of changing cement in-service lifetime to contribute to a potential reduction in cement demand was based on Miller [81]. This potential reduction in cement demand was calculated by using a normal distribution:

$$\mu = baseline, mean\ service\ lifetime \tag{60}$$

$$\sigma = 0.2 * \mu \tag{61}$$

where μ is the mean and σ is the standard deviation, based on the modeling assumption by Cao et al. [71]. In this work, the influence that elongating time to removal from in-service life was addressed.

S.6. Cumulative GHG emissions reduction potential

To quantitatively assess the reduction in GHG emissions for cement-based materials production possible from each of the strategies considered in this work, several steps of analysis were performed. To determine the annual reduction in GHG emissions per m^3 of concrete between 2015 and 2100, linear interpolation was used between the baseline value in 2015 and the greatest reduction to be achieved in 2100.

S.6.1. Projection of future cement demand

The estimated future cement demand was based on the model developed by Cao et al. [82], which work focused on production after 1950. For this 150-year period, cement inflow was captured based on a stock-driven approach. In this approach, the Gompertz combined model was applied, using the same input parameters as outlined by Cao et al. [82]:

$$S_{j,t_n} = \frac{S_{j,sat}}{1 + \left(\frac{S_{j,sat}}{S_{j,t_0}} - 1\right) \times \exp\left(A \times \left(1 - \exp\left(B \times (t_n - t_0)\right)\right)\right)} \tag{62}$$

where S_{j,t_n} is the per capita cement stock at year t_n, $S_{j,sat}$ is the per capita cement stock saturation; S_{j,t_0} is the per capita stock level at the initial time t_0; j is the sectoral fraction; and A and B refer to parameters that reflect growth patterns over time. The model was used to calculate the growth curve of future per capita cement stock based on in-service lifetimes and stock patterns developed by Cao et al. [82]. Namely, the data for saturation level (medium), saturation times (moderate) and lifetimes were based directly on Cao et al. [82]. Projected population data from the UN World Population Prospects [83] were used to capture anticipated changes in population; the medium variant was used in the analysis herein, and the low and high variants were used to analyze the impact of alternative population growth patterns between 2020-2100 [83]. The ten countries/regions modeled were: (1) North America; (2) Latin America; (3) Europe; (4) Commonwealth of Independent States (CIS); (5) China; (6) India; (7) Africa; (8) the Middle East; (9) Developed Asia & Oceania; and (10) Developing Asia. Cement demand was calculated based on inflow for each application (namely, residential, non-residential, and civil infrastructure – abbreviated as civil herein) for each of the countries/regions noted. To perform this calculation, the following equation was used:

$$\mathrm{IN}_{j,t_n} = \left(\mathrm{NET}_{j,t_n}\right) * (1 - F(t_n)) \tag{63}$$

where IN_{j,t_n} is the inflow of cement (cement demand) and NET_{j,t_n} is the net-inflow of cement each year, $(1 - F(t_n))$ is the survival function, and $F(t_n)$ is the normal cumulative distribution function at year t_n. The standard deviation for the normal distribution is set as 0.2 of the mean lifetimes.

The net-inflow of cement each year is calculated as the difference between the total cement stock in year t_n and the total cement stock the previous year:

$$\mathrm{NET}_{j,t_n} = \left(S_{j,t_n} * P_{t_n}\right) - \left(S_{j,t_{n-1}} * P_{t_{n-1}}\right) \tag{64}$$

where, P_{t_n} is the population at year t_n and S_{j,t_n} the in-use cement stock per capita.

S.6.2. GHG emissions reduction from altering production processes

The first mitigation method considered was to draw comparisons between improvements that could be implemented in the processes used for cement and concrete production. The efficacy of these improvements was examined through the mean between the three compressive strengths, which was then used to examine emissions reduction potential for year 2100. For the diagrams made in the main article, the reduction achieved if combining all of above production reduction methods was used (see Table S.4). This reduction of GHG emissions to be 79% of the baseline emissions was modeled as being achieved in the year 2100, with a linear interpolation between technology adoption between 2015 and 2100. Reductions are determined between the median GHG emissions of the baseline case and the process-related mitigation methods.

Table S.4. Fraction of GHG emissions relative to the baseline from process-related mitigation methods discussed in Section S.2. The mean value represents the mean reduction of the three strength categories.

	Cement production reduction - Median			
	20 MPa	35 MPa	50 MPa	Mean
Efficient kiln	0.986	0.986	0.986	0.986
Gas thermal	0.856	0.856	0.852	0.855
Wind	0.943	0.942	0.942	0.942
All	0.790	0.796	0.785	0.790

S.6.3. GHG emissions reduction from altering constituents

In addition to altering manufacturing processes, the role of changing constituents used, namely to increase mineral additive content, was considered as a means to reduce GHG emissions from global cement-based materials production. In order to estimate the role these mineral additives could play in lowering GHG emissions, cement replacement levels had to be approximated.

To calculate the potential reduction in environmental impact per m^3 of concrete (as well as mortar used) when using a higher ratio of cement replacement, concrete mixtures were modeled as containing a higher cement replacement ratio than the 2015 baseline (20.3%). To do this, two permutations of increased replacement were considered: one with additional pozzolanic materials (an increase up to 30% SCM content) and one with additional cementitious and pozzolanic materials (an increase up to 50% SCM content). In both of these cases, because a variety of SCMs could be used and new SCM permutations are continuously being researched [84], our models assume the environmental impacts of producing these SCMs to reach higher replacement levels were equivalent to those for quarrying/crushing natural pozzolans.

The GHG emissions reduction potential from using these higher SCM replacement ratio mixtures were compared to the 2015 average GHG emissions per m^3 of concrete (~266 kg CO_2-eq/m^3) with 20.3% cement replacement on average. A net reduction of 11.1% and 34.0% was estimated for replacement with 30% and 50% SCM replacement, respectively. As such, to determine the annual reduction in GHG emissions per m^3 of concrete between 2015 and 2100, linear interpolation between the baseline value in 2015 and potential target value, 66.0% of projected GHG emissions when higher cement replacement is used was employed.

S.6.4. The role of concrete member design

To incorporate the effects of member design on potential reduction in GHG emissions, the effects of using higher compressive strength in concrete columns for buildings was examined, as well as the effect of optimizing the reinforcement ratio and strength of slabs. This component of buildings was assessed due to the anticipated universality of findings. To determine the volume of concrete that could be reduced through use of higher compressive strength in columns of multi-story buildings, an estimated ratio of concrete used for columns relative to the slab was calculated. To perform this calculation, a 20 story RC structure consisting of four columns and a slab was assumed. Due to varying load on the columns (because the bottom columns are subjected to higher load than the top story columns for example), the average column volume was applied. For the slab, the volume of concrete required when specifying a low compressive strength (20MPa) was used for all design comparisons. Using these inputs, the resulting column-to-slab ratio is 12.0% column and 88.0% slab. The potential saving in concrete volume, and therefore GHG emissions, that can be achieved by optimizing the concrete compressive strength and reinforcement ratio (40MPa with minimum reinforcement), compared to baseline chosen as 30MPa with median reinforcement ratio (3.5%), is 24.3%, 24.1% and 20.7% for the Indian Standard, Eurocode 2 and ACI codes, respectively. In this case, a reduction in GHG emissions for reinforced concrete columns by 2100 was estimated using the following equation (using the average reduction of the design codes):

$$80.1\% * \mathrm{GHG} * 12.0\%\ \mathrm{column\ ratio} = 9.6\%\ \mathrm{GHG} \quad (64)$$

Similarly, to determine the amount of concrete (or GHG emissions) that could be saved by more efficient design of reinforced concrete slabs, a slab with median reinforcement ratio (0.25%, note that all slabs are deflection controlled and within allowable *h/l* ratio, resulting in low reinforcement ratio) was assumed as the baseline to compare with the combination of reinforcement ratio and compressive strength that results in the smallest environmental impact for a 1m strip of a reinforced concrete slab. A comparison was made between the three design codes to determine which code generates the smallest impact for a slab with the same span length, subjected to the same load. (In this case, we compared the baseline and lowest impact for ultimate design stage). The reduction in GHG emissions for the slab for the Indian Standard, Eurocode 2 and ACI were 25.2%, 24.4% and 20.9%. The total reduction, on average, in GHG emissions for reinforced concrete slabs by 2100 was estimated as:

$$76.5\% * \mathrm{GHG} * 88.0\%\ \mathrm{slab\ ratio} = 67.3\%\ \mathrm{GHG} \quad (65)$$

Therefore, the reduction by year 2100 by design optimization of reinforced concrete columns and slabs was estimated to:

$$0.096 + 0.673 = 0.769 \quad (66)$$

of the baseline emissions. Note that this only applies to 80% of the total emissions from concrete used for building structural applications (~18% reduction of the total global emissions from cement-based materials), as it was assumed that 20% of concrete is used for other applications than columns and slabs.

S.6.5. The role of elongating concrete service life

To examine the influence of elongating concrete service life, if elongating service life could be used to offset new cement-based materials production, three scenarios were considered. Namely, herein we consider the effects of:

1. The "ideal" scenario – here, all countries/regions of the world are modeled as being able to use up to 50% SCM replacement of Portland cement, and that increase in SCM use could lead to a potential service life extension of up to 4-fold for Res, NonRes and CE. Further, this extension of service life is applied to historic (before 2015) concrete structures, based on the assumption that improvements to cementitious binders have been growing over time.
2. The "realistic" scenario – here, it is assumed that certain parts of the world may not have easy access to SCMs, which could limit the abilities to achieve 50% Portland cement replacement uniformly. As such, it is assumed that only 50% of the world would have access to enough SCMs to meet this replacement ratio, and it is further assumed that only CE structures can reach 4-fold service life extension with buildings (both Res and NonRes) achieving a 3-fold service life extension. Again, here this extension of service life is applied to historic (before 2015) concrete structures, based on the assumption that improvements to cementitious binders have been growing over time.
3. The "future" scenario – here, the assumptions from the "realistic" scenario were considered as only applicable for future concrete structures (i.e., after 2015) and no elongated service life beyond historic trends could be expected for any in-use cement-based materials.

In this counterfactual model, the assumption is made that if structures can last for a longer period of time, it could offset the production of more cement to establish structures of similar purpose. Here, it is found that percent reduced cement demand from elongating service life would be large in regions with shorter service-periods (see Tables S.5 – S.7).

Table S.5. Reduction in cement demand (Mt) and greenhouse gas (GHG) emissions (Gt) for "ideal scenario". (Buildings include both residential (Res) and non-residential (NonRes)) between 1931-2100. CIS = Commonwealth of Independent States.

**Ideal Scenario**	Cement Reduced [Mt]			GHG Emissions Reduced [Gt]			% Reduction		
Country/region:	Buildings	Civil	Total	Buildings	Civil	Total	Buildings	Civil	Total
India	24447.2	12613.3	37060.5	20.7	10.7	31.4	56.1%	56.7%	56.3%
Africa	13999.5	21352.1	35351.6	11.8	18.1	29.9	21.1%	43.0%	30.4%
Europe	8115.3	7017.3	15132.6	6.9	5.9	12.8	52.5%	68.0%	58.7%
Latin America	5924.0	2665.1	8589.1	5.0	2.3	7.3	39.8%	36.9%	38.9%
North America	4137.0	3710.9	7848.0	3.5	3.1	6.6	37.5%	52.0%	43.2%
Middle East	13879.1	6787.9	20667.0	11.7	5.7	17.5	65.1%	65.2%	65.1%
China	70256.2	32392.8	102649.0	59.5	27.4	86.9	76.5%	76.3%	76.5%
Developed Asia & Oceania	8596.7	5760.3	14357.0	7.3	4.9	12.2	71.8%	76.5%	73.6%
Developing Asia	23323.7	12691.3	36015.0	19.7	10.7	30.5	55.6%	57.3%	56.2%
CIS	4456.4	3756.7	8213.1	3.8	3.2	7.0	52.0%	64.5%	57.1%
World	177135.1	108747.7	285882.8	149.9	92.1	242.0	54.2%	58.8%	55.8%

Table S.6. Reduction in cement demand (Mt) and greenhouse gas (GHG) emissions (Gt) for "realistic scenario". (Buildings include both residential (Res) and non-residential (NonRes)) between 1931-2100. CIS = Commonwealth of Independent States.

**Realistic Scenario**	Cement Reduced [Mt]			GHG Emissions Reduced [Gt]			% Reduction		
Country/region:	Buildings	Civil	Total	Buildings	Civil	Total	Buildings	Civil	Total
India	8838.5	8404.3	17242.8	7.7	7.3	14.9	53.5%	40.7%	27.9%
Africa	9422.3	16296.2	25718.5	8.2	14.1	22.3	18.1%	33.8%	22.9%
Europe	1173.8	1602.2	2776.0	1.0	1.4	2.4	16.2%	32.4%	22.3%
Latin America	2782.5	836.0	3618.5	2.4	0.7	3.1	24.9%	15.7%	20.9%
North America	680.8	1187.7	1868.5	0.6	1.0	1.6	9.2%	25.5%	15.4%
Middle East	3541.5	3444.2	6985.7	3.1	3.0	6.1	60.0%	40.3%	25.9%
China	12639.4	13034.5	25673.9	11.0	11.3	22.2	66.7%	35.4%	24.1%
Developed Asia & Oceania	1753.5	1948.0	3701.6	1.5	1.7	3.2	54.8%	42.6%	30.5%
Developing Asia	8178.6	8123.0	16301.6	7.1	7.0	14.1	52.5%	39.9%	27.4%
CIS	1592.4	950.7	2543.1	1.4	0.8	2.2	29.7%	31.3%	28.4%
World	50603.4	55826.8	106430.2	43.8	48.4	92.2	44.9%	35.5%	24.7%

Table S.7. Reduction in cement demand (Mt) and greenhouse gas (GHG) emissions (Gt) for "future scenario". (Buildings include both residential (Res) and non-residential (NonRes)) between 1931-2100. CIS = Commonwealth of Independent States.

**Future Scenario**	Cement Reduced [Mt]			GHG Emissions Reduced [Gt]			% Reduction		
Country/region:	Buildings	Civil	Total	Buildings	Civil	Total	Buildings	Civil	Total
India	22051.0	10985.4	33036.4	19.1	9.5	28.6	53.5%	53.1%	53.4%
Africa	11576.3	19768.5	31344.8	10.0	17.1	27.2	18.1%	41.1%	27.9%
Europe	1209.3	1603.1	2812.4	1.0	1.4	2.4	16.2%	32.4%	22.6%
Latin America	2994.0	836.0	3830.0	2.6	0.7	3.3	24.9%	15.7%	22.1%
North America	688.3	1187.9	1876.2	0.6	1.0	1.6	9.2%	25.5%	15.5%
Middle East	11078.2	4905.4	15983.5	9.6	4.2	13.8	60.0%	57.3%	59.2%
China	46676.8	26122.4	72799.3	40.4	22.6	63.1	66.7%	71.0%	68.2%
Developed Asia & Oceania	4150.0	2859.5	7009.5	3.6	2.5	6.1	54.8%	62.6%	57.8%
Developing Asia	20601.6	10824.6	31426.2	17.8	9.4	27.2	52.5%	53.2%	52.7%
CIS	1757.5	950.9	2708.4	1.5	0.8	2.3	29.7%	31.3%	30.2%
World	122783.2	80043.7	202826.9	106.4	69.3	175.7	44.9%	51.0%	47.1%

As was done with the other mitigation methods, the influence of an increase in concrete longevity on reducing cement demand, and the associated GHG emissions from new material production, linear interpolation between 2015 baseline value and reduction due to in-service life increase as per the 'future' scenario described below. The reduction in cement demand estimated for 2100 was 47.1%, corresponding to 52.9% of materials required relative to if no modifications had been made (up to 4-fold lifetime increase, see Table S.7 for the estimated total reduction for the World). If including the reduced cement demand due to higher use of SCMs (50%) to achieve the increased service-life, the reduction in cement demand estimated for 2100 was 65%.

For the 3 scenarios modeled:

- 1. Ideal Scenario: SCMs are available so that 50% cement replacement can be achieved all over the world, and the service-lifetime of buildings (Res and NonRes) and Civil can as a result be extended by 4-fold. Lifetime extension has been applied to historic and future concrete structures (built between 1931-2100)
- For the ideal scenario, 285.9 Gt cement can be reduced, which is equivalent to 242.0 Gt GHG (55.8% reduction) emissions.
- 2. Future Only Scenario: SCMs are available so that 50% cement replacement can be achieved all over the world, but the service life is only extended for concrete structures built after 2015. The service-lifetime of buildings (Res and NonRes) can be extended by 3-fold and infrastructure (CE) can be extended by 4-fold.
- For the future scenario, 202.8 Gt cement can be reduced, which is equivalent to 175.7 Gt emissions (47.1% reduction).
- For future scenario, but with 30% replacement, the service-life of buildings (Res and NonRes) can be extended by 2-fold and infrastructure (Civile) can be extended by 3-fold. For this scenario, 167.4 Gt cement can be reduced, which is equivalent to 145.1 Gt emissions (38.9% reduction).

- 3. Realistic Scenario: Due to limited accessibility to SCMs in some countries/regions, we assume that 50% cement replacement can be achieved for 50% of all concrete structures built after 2015, and the service-lifetime of buildings (Res and NonRes) can be extended by 3-fold and infrastructure (Civil) can be extended by 4-fold.
- For the realistic scenario, 106.4 Gt cement can be reduced, which is equivalent to 92.2 Gt emissions (24.7% reduction).

The results from the sensitivity analysis, where the impact of lower and higher population growth was examined, are shown in Table S.8.

Table S.8. Sensitivity analysis for reduction in cement (Mt) and greenhouse gas (GHG) emissions (Gt) for three scenarios of population growth between 2020-2100; low, medium, and high. The "future" scenario for 50% cement replacement is assumed.

Future Scenario - Cement [Mt]			
Scenario:	Demand	Reduced	Reduction
Low	331009.8	176962.0	53.5%
Medium	430459.5	202826.9	47.1%
High	557262.2	231709.8	41.6%
**Future Scenario - GHG Emissions [Gt]**			
Scenario:	Demand	Reduced	Reduction
Low	286.8	153.3	53.5%
Medium	372.9	175.7	47.1%
High	482.8	200.8	41.6%

Figure S1. Potential reduction in greenhouse gas (GHG) emissions between 2015-2100 for (a) low variant population growth and (b) high variant population growth. For the median variant, see Figure 4 in the manuscript.

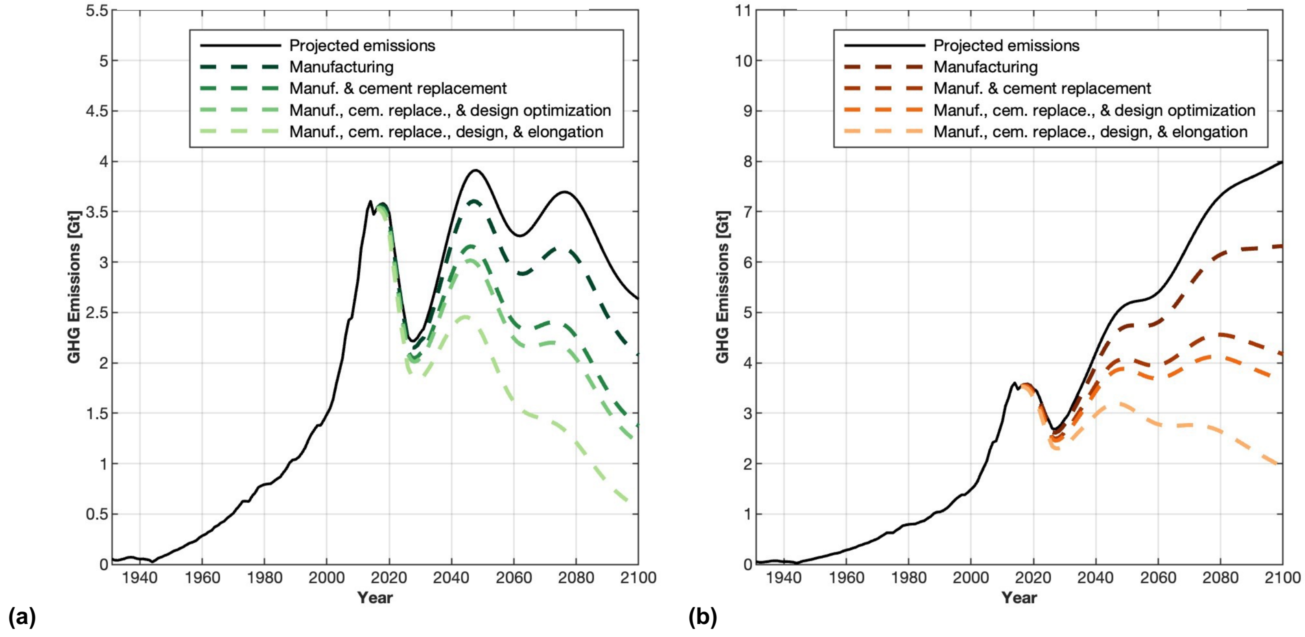

S.6.6. The potential influence of building height

While not a direct part of the GHG emissions mitigation strategies addressed in this work, it has been posited that building height could be a strong influencer on the environmental impacts of the materials selected for the structural design (note: contributions from varying foundations, roofs, and other materials beyond the frame are excluded from consideration) [85]. To conduct an initial examination into the role building height could play in reducing GHG emissions from concrete structures, the effects of stacked versus unstacked units were examined herein (see Section S.3.3).

Using each of the three building codes applied in this work, namely the ACI code, the Eurocode 2, and the Indian Standard, approximate shifts in GHG emissions to produce stacked versus non-stacked structures were estimated. The GHG emissions trends are shown in Figures S1-S3.

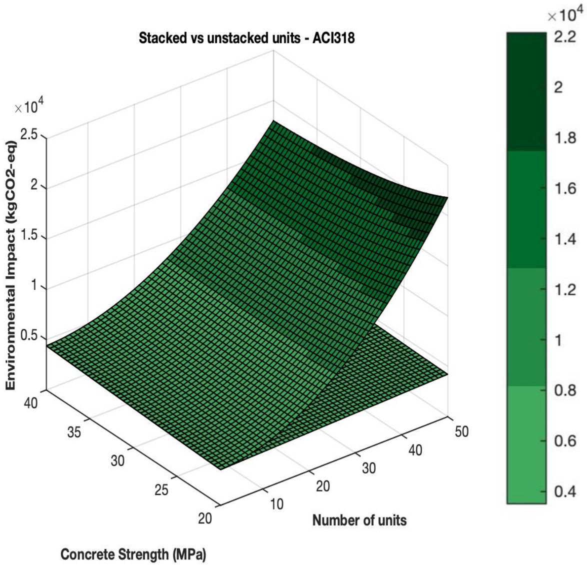

Figure S2. GHG emissions from production for varying number of units stacked (upper surface) or unstacked (bottom surface) when designing according to ACI318.

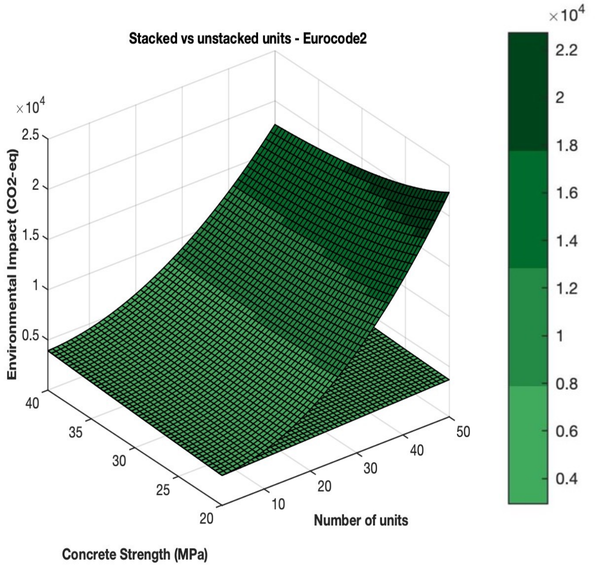

Figure S3. GHG emissions from production for varying number of units stacked (upper surface) or unstacked (bottom surface) when designing according to EuroCode2.

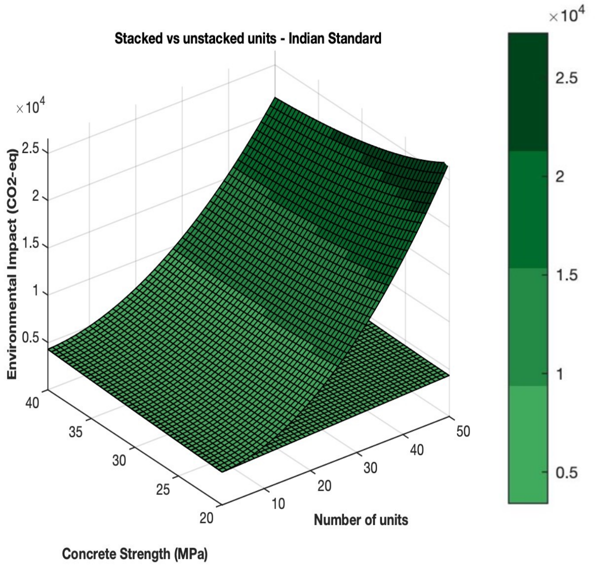

Figure S4. GHG emissions from production for varying number of units stacked (upper surface) or unstacked (bottom surface) when designing according to IS 456:2000.

The figures above show that designing buildings to be shorter (stacking fewer units, thus having fewer building stories) has the potential to be beneficial in reducing GHG emissions. This result is due less load on concrete columns, and therefore less volume of concrete required to sustain the loads. However, there are limited data on the average building height (globally or for certain countries/regions). Thus, it is difficult to scale these results in order to estimate the mitigation of GHG emissions if shorter buildings than average would be built in the future (or at least not taller since we are likely heading in that direction). Further, these models to do not incorporate factors such as the amount of material needed for roofs, foundations, or other aspects that would vary based on “stacking” structural units versus

"unstacking" these units and placing them side-by-side. As such, this is considered to be an area for future study, but outside the scope of this work.

Supplementary Information References

[1] D.R. Gomez, J.D. Watterson, B.B. Americano, C. Ha, G. Marland, E. Matsika, L.N. Namayanga, B. Osman-Elasha, J.D.K. Saka, K. Treanton, R. Quadrelli, I.P. on C. Change, 2006 IPCC Guidelines for National Greenhouse Gas Inventories: Chapter 2: Energy: Stationary Combustion, Intergovernmental Panel on Climate Change, Hayama, Kanagawa, 2007.

[2] J. Rissman, C. Bataille, E. Masanet, N. Aden, W.R. Morrow, N. Zhou, N. Elliott, R. Dell, N. Heeren, B. Huckestein, J. Cresko, S.A. Miller, J. Roy, P. Fennell, B. Cremmins, T. Koch Blank, D. Hone, E.D. Williams, S. de la Rue du Can, B. Sisson, M. Williams, J. Katzenberger, D. Burtraw, G. Sethi, H. Ping, D. Danielson, H. Lu, T. Lorber, J. Dinkel, J. Helseth, Technologies and policies to decarbonize global industry: Review and assessment of mitigation drivers through 2070, Appl. Energy. 266 (2020) 114848. https://doi.org/10.1016/j.apenergy.2020.114848.

[3] A. Kim, P.R. Cunningham, K. Kamau-Devers, S.A. Miller, OpenConcrete: a tool for estimating the environmental impacts from concrete production, Environ. Res. Infrastruct. Sustain. (2022). https://doi.org/10.1088/2634-4505/ac8a6d.

[4] A. Kim, P.R. Cunningham, K. Kamau-Devers, S.A. Miller, OpenConcrete: An open-source tool for estimating the environmental impacts from concrete production, Environ. Sci. Technol. (n.d.).

[5] GNR, Global Cement Database on CO_2 and Energy Information, 2016 (2016).

[6] GCCA, GNR – GCCA in Numbers, Glob. Cem. Concr. Assoc. (2020).

[7] IEA, Data and Statistics, (2020).

[8] K. Scrivener, F. Martirena, S. Bishnoi, S. Maity, Calcined clay limestone cements (LC3), Cem. Concr. Res. 114 (2018) 49–56. https://doi.org/10.1016/j.cemconres.2017.08.017.

[9] I.H. Shah, S.A. Miller, D. Jiang, R.J. Myers, Cement substitution with secondary materials can reduce annual global CO2 emissions by up to 1.3 gigatons, Nat. Commun. 13 (2022) 1–11. https://doi.org/10.1038/s41467-022-33289-7.

[10] C. Bilim, C.D. Atiş, H. Tanyildizi, O. Karahan, Predicting the compressive strength of ground granulated blast furnace slag concrete using artificial neural network, Adv. Eng. Softw. 40 (2009) 334–340. https://doi.org/http://dx.doi.org/10.1016/j.advengsoft.2008.05.005.

[11] A. Oner, S. Akyuz, R. Yildiz, An experimental study on strength development of concrete containing fly ash and optimum usage of fly ash in concrete, Cem. Concr. Res. 35 (2005) 1165–1171. https://doi.org/10.1016/j.cemconres.2004.09.031.

[12] R. Liu, S. Durham, K. Rens, A. Ramaswami, Optimization of Cementitious Material Content for Sustainable Concrete Mixtures, J. Mater. Civ. Eng. 24 (2012) 745–753. https://doi.org/doi:10.1061/(ASCE)MT.1943-5533.0000444.

[13] K. Celik, C. Meral, A.P. Gursel, P.K. Mehta, A. Horvath, P.J.M. Monteiro, Mechanical properties, durability, and life-cycle assessment of self-consolidating concrete mixtures made with blended portland cements containing fly ash and limestone powder, Cem. Concr. Compos. 56 (2015) 59–72. https://doi.org/10.1016/j.cemconcomp.2014.11.003.

[14] M. Seddik Meddah, Durability performance and engineering properties of shale and volcanic ashes concretes, Constr. Build. Mater. 79 (2015) 73–82. https://doi.org/10.1016/j.conbuildmat.2015.01.020.

[15] M.S. Meddah, M.C. Lmbachiya, R.K. Dhir, Potential use of binary and composite limestone cements in concrete production, Constr. Build. Mater. 58 (2014) 193–205. https://doi.org/10.1016/j.conbuildmat.2013.12.012.

[16] S.E. Hedegaard, T.C. Hansen, Modified water/cement ratio law for compressive strength of fly ash concretes, Mater. Struct. 25 (1992) 273–283. https://doi.org/10.1007/bf02472668.
[17] B. Felekoğlu, S. Türkel, B. Baradan, Effect of water/cement ratio on the fresh and hardened properties of self-compacting concrete, Build. Environ. 42 (2007) 1795–1802. https://doi.org/http://dx.doi.org/10.1016/j.buildenv.2006.01.012.
[18] E. Vejmelková, M. Pavlíková, Z. Keršner, P. Rovnaníková, M. Ondráček, M. Sedlmajer, R. Černý, High performance concrete containing lower slag amount: A complex view of mechanical and durability properties, Constr. Build. Mater. 23 (2009) 2237–2245. https://doi.org/http://dx.doi.org/10.1016/j.conbuildmat.2008.11.018.
[19] M.N. Haque, O. Kayali, Properties of high-strength concrete using a fine fly ash, Cem. Concr. Res. 28 (1998) 1445–1452. https://doi.org/http://dx.doi.org/10.1016/S0008-8846(98)00125-2.
[20] K.-R. Wu, B. Chen, W. Yao, D. Zhang, Effect of coarse aggregate type on mechanical properties of high-performance concrete, Cem. Concr. Res. 31 (2001) 1421–1425. https://doi.org/http://dx.doi.org/10.1016/S0008-8846(01)00588-9.
[21] M.S. Meddah, M.A. Ismail, S. El-Gamal, H. Fitriani, Performances evaluation of binary concrete designed with silica fume and metakaolin, Constr. Build. Mater. 166 (2018) 400–412. https://doi.org/10.1016/j.conbuildmat.2018.01.138.
[22] R. Siddique, Performance characteristics of high-volume Class F fly ash concrete, Cem. Concr. Res. 34 (2004) 487–493. https://doi.org/http://dx.doi.org/10.1016/j.cemconres.2003.09.002.
[23] A. Oner, S. Akyuz, An experimental study on optimum usage of GGBS for the compressive strength of concrete, Cem. Concr. Compos. 29 (2007) 505–514. https://doi.org/10.1016/j.cemconcomp.2007.01.001.
[24] C.S. Poon, L. Lam, Y.L. Wong, A study on high strength concrete prepared with large volumes of low calcium fly ash, Cem. Concr. Res. 30 (2000) 447–455. https://doi.org/http://dx.doi.org/10.1016/S0008-8846(99)00271-9.
[25] L. Lam, Y.L. Wong, C.S. Poon, Effect of Fly Ash and Silica Fume on Compressive and Fracture Behaviors of Concrete, Cem. Concr. Res. 28 (1998) 271–283. https://doi.org/http://dx.doi.org/10.1016/S0008-8846(97)00269-X.
[26] A.A. Ramezanianpour, V.M. Malhotra, Effect of curing on the compressive strength, resistance to chloride-ion penetration and porosity of concretes incorporating slag, fly ash or silica fume, Cem. Concr. Compos. 17 (1995) 125–133. https://doi.org/https://doi.org/10.1016/0958-9465(95)00005-W.
[27] C.-H. Huang, S.-K. Lin, C.-S. Chang, H.-J. Chen, Mix proportions and mechanical properties of concrete containing very high-volume of Class F fly ash, Constr. Build. Mater. 46 (2013) 71–78. https://doi.org/https://doi.org/10.1016/j.conbuildmat.2013.04.016.
[28] P. Chindaprasirt, S. Homwuttiwong, C. Jaturapitakkul, Strength and water permeability of concrete containing palm oil fuel ash and rice husk–bark ash, Constr. Build. Mater. 21 (2007) 1492–1499. https://doi.org/https://doi.org/10.1016/j.conbuildmat.2006.06.015.
[29] A. Behnood, H. Ziari, Effects of silica fume addition and water to cement ratio on the properties of high-strength concrete after exposure to high temperatures, Cem. Concr. Compos. 30 (2008) 106–112. https://doi.org/https://doi.org/10.1016/j.cemconcomp.2007.06.003.
[30] K.-S. Youm, J. Moon, J.-Y. Cho, J.J. Kim, Experimental study on strength and durability of lightweight aggregate concrete containing silica fume, Constr. Build. Mater. 114 (2016) 517–527. https://doi.org/https://doi.org/10.1016/j.conbuildmat.2016.03.165.
[31] S.-T. Yi, E.-I. Yang, J.-C. Choi, Effect of specimen sizes, specimen shapes, and placement directions on compressive strength of concrete, Nucl. Eng. Des. 236 (2006) 115–127. https://doi.org/http://dx.doi.org/10.1016/j.nucengdes.2005.08.004.

[32] C. Fan, S.A. Miller, Reducing greenhouse gas emissions for prescribed concrete compressive strength, Constr. Build. Mater. 167 (2018) 918–928. https://doi.org/10.1016/j.conbuildmat.2018.02.092.
[33] P. Kourehpaz, S.A. Miller, Eco-efficient design indices for reinforced concrete members, n.d.
[34] A.C. I, 318-11: Building Code Requirements for Structural Concrete, (2011).
[35] CEN, Eurocode 2: Design of concrete structures - Part 1-1: General rules and rules for buildings (EN 1992-1-1 (2004)), Brussels, Belgium, 2004.
[36] ICS, IS 456 (2000): Plain and Reinforced Concrete - Code ofPractice, New Dehli, India, 2007.
[37] E. Commission, Joint Research Centre, (2017).
[38] A. Kapur, G. Keoleian, A. Kendall, S.E. Kesler, Dynamic modeling of in-use cement stocks in the United States, J. Ind. Ecol. 12 (2008) 539–556. https://doi.org/10.1111/j.1530-9290.2008.00055.x.
[39] W. Schmidt, J. Anniser, K. Manful, A sustainability point of view on horizontal and vertical urban growth, in: W. Schmidt (Ed.), ISEE Africa Innov. Sci. Eng. Educ., Bundesanstalt für Materialforschung und -prüfung (BAM), Nairobi, Kenya, 2019: pp. 189–193.
[40] ACI, Building Code Requirements for Structural Concrete (ACI 318-14), Farmington Hills, MI, 2014.
[41] M. Alexander, H. Beushausen, Durability, service life prediction, and modelling for reinforced concrete structures – review and critique, Cem. Concr. Res. 122 (2019) 17–29. https://doi.org/10.1016/j.cemconres.2019.04.018.
[42] A. Bentur, Steel Corrosion in Concrete: Fundamentals and civil engineering practice, 1st ed., CRC Press, 1997. https://doi.org/10.1201/9781482271898.
[43] U. Angst, F. Moro, M. Geiker, S. Kessler, H. Beushausen, C. Andrade, J. Lahdensivu, A. Köliö, K.I. Imamoto, S. von Greve-Dierfeld, M. Serdar, Corrosion of steel in carbonated concrete: Mechanisms, practical experience, and research priorities – A critical review by RILEM TC 281-CCC, RILEM Tech. Lett. 5 (2020) 85–100. https://doi.org/10.21809/rilemtechlett.2020.127.
[44] J.R. Mackechnie, M.G. Alexander, A rational design approach for durable marine concrete structures, J. South African Inst. Civ. Eng. 39 (1997) 11–15.
[45] J.R. Mackechnie, M.G. Alexander, Exposure of concrete in different marine environments, J. Mater. Civ. Eng. 9 (1997) 41–44. https://doi.org/10.1061/(ASCE)0899-1561(1997)9:1(41).
[46] Z. Shi, B. Lothenbach, M.R. Geiker, J. Kaufmann, A. Leemann, S. Ferreiro, J. Skibsted, Experimental studies and thermodynamic modeling of the carbonation of Portland cement, metakaolin and limestone mortars, Cem. Concr. Res. 88 (2016) 60–72. https://doi.org/10.1016/j.cemconres.2016.06.006.
[47] O. Bowles, B.W. Bagley, A.T. Coons, Cement, in: H.H. Hughes (Ed.), Miner. Yearb. 1937, United States Government Printing Office, Washington D.C., 1937.
[48] O. Bowles, E. V. Balser, Cement, in: E.W. Pehrson, H.D. Keiser (Eds.), Miner. Yearb. Rev. 1940, United States Government Printing Office, Washington D.C., 1941.
[49] B.C. Brown, Cement, in: Miner. Yearb. Met. Miner. Fuels 1974, U.S. Bureau of Mines, Washington D.C., 1974.
[50] E.G. Hoover, Cement, in: Miner. Yearb. Met. Miner. 1977, U.S. Bureau of Mines, Washington D.C., 1977.
[51] R.H. Singleton, C.L. Davis, Cement, in: Miner. Yearb. Met. Miner. 1980, U.S. Bureau of Mines, Washington D.C., 1980.
[52] W. Johnson, S.T. Absalom, Cement, in: Miner. Yearb. Met. Miner. 1982, U.S. Bureau of Mines, Washington D.C., n.d.
[53] W. Johnson, Cement, in: Miner. Yearb. Met. Miner. 1985, U.S. Bureau of Mines, Washington

D.C., 1985.
[54] W. Johnson, Cement, in: Miner. Yearb. 1988, U.S. Bureau of Mines, Washington D.C., 1988.
[55] C. Solomon, Cement, in: Miner. Yearb. 1991, U.S. Bureau of Mines, Washington D.C., 1991.
[56] C. Solomon, Cement, in: Miner. Yearb., United States Geological Survey, Reston, VA, 1994.
[57] H.G. van Oss, Cement, in: Miner. Yearb., United States Geological Survey, Reston, VA, 1998.
[58] H.G. van Oss, Cement, in: Miner. Yearb., United States Geological Survey, Reston, VA, 2002.
[59] O. Bowles, E. V. Balser, Cement, in: E.W. Pehrson, H.D. Keiser (Eds.), Miner. Yearb. 1945, United States Government Printing Office, Washington D.C., 1947.
[60] H.G. van Oss, 2006 Minerals Yearbook: Cement, in: Miner. Yearb., United States Geological Survey, Reston, VA, 2008.
[61] H.G. van Oss, 2010 Minerals Yearbook: Cement, in: Miner. Yearb., United States Geological Survey, Reston, VA, 2012.
[62] H.G. van Oss, 2014 Minerals Yearbook: Cement [Advance Release], in: Miner. Yearb., United States Geological Survey, Reston, VA, 2017.
[63] H.G. van Oss, 2015 Minerals Yearbook: Cement [Advance Release], in: Miner. Yearb., United States Geological Survey, Reston, VA, 2018.
[64] O.S. North, E. V. Balser, Cement, in: L.L. Fischman (Ed.), Miner. Yearb. 1950, Bureau of Mines, United States Government Printing Office, Washington D.C., 1953.
[65] D.O. Kennedy, B.M. Moore, Cement, in: Miner. Yearb. Met. Miner. (except Fuels) 1954, Bureau of Mines, United States Government Printing Office, Washington D.C., 1958.
[66] D.O. Kennedy, A.H. Lindquist, Cement, in: Miner. Yearb. Met. Miner. (except Fuels) 1959, Bureau of Mines, United States Government Printing Office, Washington D.C., 1960.
[67] W.R. Barton, Cement, in: Miner. Yearb. Met. Miner. (except Fuels) 1964, Bureau of Mines, United States Government Printing Office, Washington, D.C., 1965.
[68] J.R. Lewis, Cement, in: Miner. Yearb. Met. Miner. Fuels 1968, Bureau of Mines, United States Government Printing Office, Washington D.C., 1969.
[69] B.C. Brown, Cement, in: Miner. Yearb. Met. Miner. Fuels 1970, U.S. Bureau of Mines, Washington D.C., 1970.
[70] B.C. Brown, Cement, in: Miner. Yearb. Met. Miner. Fuels 1972, U.S. Bureau of Mines, Washington D.C., 1972.
[71] Z. Cao, L. Shen, A.N. Løvik, D.B. Müller, G. Liu, Elaborating the History of Our Cementing Societies: An in-Use Stock Perspective, Environ. Sci. Technol. 51 (2017) 11468–11475. https://doi.org/10.1021/acs.est.7b03077.
[72] U. N, UN Comtrade Database: Cement (portland, aluminous, slag or hydraulic), 2015 (2015).
[73] ERMCO, Ready-mixed Concrete Industry Statistics Year 2001, Brussels, Belgium, 2002.
[74] ERMCO, Ready-mixed Concrete Industry Statistics Year 2006, Brussels, Belgium, 2007.
[75] ERMCO, Ready-mixed Concrete Industry Statistics Year 2011, Brussels, Belgium, 2012.
[76] ERMCO, Ready-mixed Concrete Industry Statistics Year 2016, Brussels, Belgium, 2017.
[77] F. Xi, S.J. Davis, P. Ciais, D. Crawford-Brown, D. Guan, C. Pade, T. Shi, M. Syddall, J. Lv, L. Ji, L. Bing, J. Wang, W. Wei, K.-H. Yang, B. Lagerblad, I. Galan, C. Andrade, Y. Zhang, Z. Liu, Substantial global carbon uptake by cement carbonation, Nat. Geosci. 9 (2016) 880–883. https://doi.org/10.1038/ngeo2840.
[78] S.A. Miller, A. Horvath, P.J.M. Monteiro, Impacts of booming concrete production on water resources worldwide, Nat. Sustain. 1 (2018). https://doi.org/10.1038/s41893-017-0009-5.
[79] ASTM, ASTM C270 - 19a1: Standard Specification for Mortar for Unit Masonry, ASTM International, West Conshohoken, Pennsylvania, 2019.
[80] V.S. Dubovoy, J.W. Ribar, P.C. Association, Masonry Cement Mortars - A Laboratory

Investigation, 1990.
[81] S.A. Miller, The role of cement service-life on the efficient use of resources, Environ. Res. Lett. 15 (2020). https://doi.org/10.1088/1748-9326/ab639d.
[82] Z. Cao, R.J. Myers, R.C. Lupton, H. Duan, R. Sacchi, N. Zhou, T.R. Miller, J.M. Cullen, Q. Ge, G. Liu, The sponge effect and carbon emission mitigation potentials of the global cement cycle, Nat. Commun. 11 (2020) 3777. https://doi.org/10.1038/s41467-020-17583-w.
[83] UNPD, World Population Prospects 2019, Online Edition. Rev. 1., United Nations, Dep. Econ. Soc. Aff. Popul. Div. (2019).
[84] R. Snellings, Assessing, Understanding and Unlocking Supplementary Cementitious Materials, RILEM Tech. Lett. 1 (2016) 50. https://doi.org/10.21809/rilemtechlett.2016.12.
[85] W. Schmidt, M. Otieno, K. Olonade, N. Radebe, H. Van-Damme, P. Tunji-Olayeni, S. Kenai, A.T. Tawiah, K. Manful, A. Akinwale, R. Mbugua, A. Rogge, Innovation potentials for construction materials with specific focus on the challenges in Africa , RILEM Tech. Lett. 5 (2020). https://doi.org/10.21809/rilemtechlett.2020.112.
