# Supplementary material for: Near-term pathways for decarbonizing global concrete production
Source: Nat Commun. 2023 Jul 29;14:4574. doi: 10.1038/s41467-023-40302-0 (PMC10387082; doi:10.1038/s41467-023-40302-0)
Supplement: Supplementary file 3 — Description of Additional Supplementary Files [file 41467_2023_40302_MOESM3_ESM.pdf]

Description of Additional Supplementary Files

File Name: Supplementary Data 1
Description: This supplementary data file contains the data used to make the figures in the primary body of work and in the Supplementary Information pdf file.
